# Non-coding RNAs and glioma: Focus on cancer stem cells

**DOI:** 10.1016/j.omto.2022.09.005

**Published:** 2022-09-17

**Authors:** Ali Rajabi, Mehrdad Kayedi, Shiva Rahimi, Fatemeh Dashti, Seyed Mohammad Ali Mirazimi, Mina Homayoonfal, Seyed Mohammad Amin Mahdian, Michael R. Hamblin, Omid Reza Tamtaji, Ali Afrasiabi, Ameneh Jafari, Hamed Mirzaei

**Affiliations:** 1 School of Medicine, Kashan University of Medical Sciences, Kashan, Iran; 2 Student Research Committee, Kashan University of Medical Sciences, Kashan, Iran; 3 Department of Radiology, Shiraz University of Medical Sciences, Shiraz, Iran; 4 School of Medicine,Fasa University of Medical Sciences, Fasa, Iran; 5 Research Center for Biochemistry and Nutrition in Metabolic Diseases, Institute for Basic Sciences, Kashan University of Medical Sciences, Kashan, Iran; 6 Department of Pharmaceutical Nanotechnology, Faculty of Pharmacy, Tehran University of Medical Sciences, Tehran, Iran; 7 Laser Research Centre, Faculty of Health Science, University of Johannesburg, Doornfontein 2028, South Africa; 8 Electrophysiology Research Center, Neuroscience Institute, Tehran University of Medical Sciences, Tehran, Iran; 9 Department of Physiology, School of Medicine, Tehran University of Medical Sciences, Tehran, Iran; 10 Department of Internal Medicine, Iran University of Medical Sciences, Tehran, Iran; 11 Advanced Therapy Medicinal Product (ATMP) Department, Breast Cancer Research Center, Motamed Cancer Institute, ACECR, Tehran, Iran; 12 Proteomics Research Center, Shahid Beheshti University of Medical Sciences, Tehran, Iran

**Keywords:** MT: Non-coding RNAs, Glioma, Cancer stem cells

## Abstract

Glioblastoma and gliomas can have a wide range of histopathologic subtypes. These heterogeneous histologic phenotypes originate from tumor cells with the distinct functions of tumorigenesis and self-renewal, called glioma stem cells (GSCs). GSCs are characterized based on multi-layered epigenetic mechanisms, which control the expression of many genes. This epigenetic regulatory mechanism is often based on functional non-coding RNAs (ncRNAs). ncRNAs have become increasingly important in the pathogenesis of human cancer and work as oncogenes or tumor suppressors to regulate carcinogenesis and progression. These RNAs by being involved in chromatin remodeling and modification, transcriptional regulation, and alternative splicing of pre-mRNA, as well as mRNA stability and protein translation, play a key role in tumor development and progression. Numerous studies have been performed to try to understand the dysregulation pattern of these ncRNAs in tumors and cancer stem cells (CSCs), which show robust differentiation and self-regeneration capacity. This review provides recent findings on the role of ncRNAs in glioma development and progression, particularly their effects on CSCs, thus accelerating the clinical implementation of ncRNAs as promising tumor biomarkers and therapeutic targets.

## Graphical abstract

**Figure:**
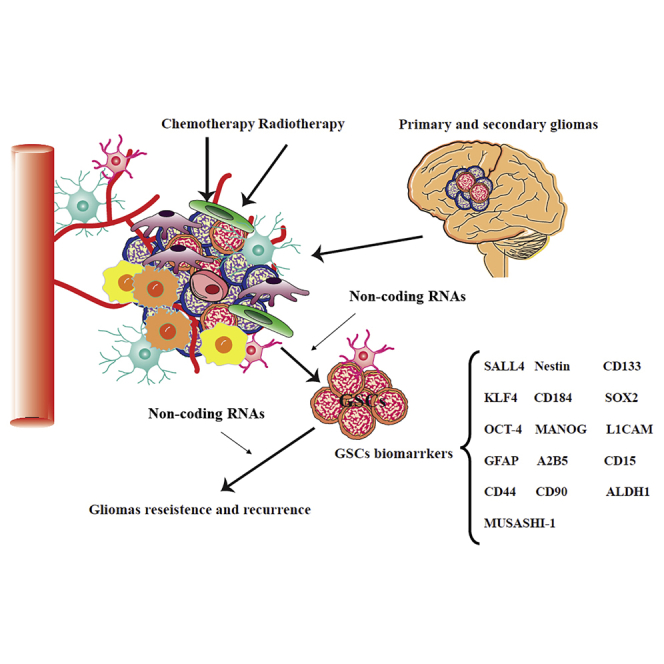

## Teaser

The presence of cancer stem cells in a tumor causes metastasis to spread more readily. NcRNAs can regulate cellular signaling in CSCs and glioma cells. However, more research into the exact pathways and mechanisms of action of ncRNAs in CSCs and glioma, is required to develop more effective therapy for glioma.

## Introduction

Gliomas are the most common primary type of adult brain cancer, consisting of up to 80% of malignant brain tumors.^1^ While being the most frequent primary brain tumor, glioblastoma (GBM) is a type of glioma accounting for 57.3% of these tumors and has the worst prognosis: WHO grade IV.^1^^,^^2^ Gliomas are divided into two distinct categories. Firstly, the IDH wild-type tumor or *de novo* primary GBM, which is most commonly found in older patients (≥62 years), accounts for about 90% of all GBMs. Secondly, the IDH mutant type or secondary GBM, which more frequently occurs in 40- to 50-year-old patients and accounts for only 10% of cases. IDH mutant tumors arise from underlying low-grade astrocytomas.^2^^,^^3^

Microarrays and next-generation sequencing technology have led to significant advances in whole-genome sequencing and provided a more comprehensive understanding of non-coding RNAs (ncRNAs) and their roles and functions. The majority of the human genome (>90%) undergoes transcription, but many of these genes do not result in the synthesis of new proteins.^4^ Several ncRNAs have important regulatory functions. ncRNAs, including lncRNAs (long ncRNAs), miRs (microRNAs), and circRNAs (circular RNAs) play critical roles in numerous cell processes and are regulated by specific molecular mechanisms.^5^,^6^ miRs are a group of short endogenous ncRNAs that regulate the post-transcriptional expression of many genes.^7^ miRs are involved in many pathological and physiological cellular processes, including tumorigenesis and cancer progression. Dysregulated miRNA expression may result either in tumor inhibition or in tumor promotion as an oncogene.^8^^,^^9^ circRNAs are a class of ncRNA with covalently closed loops and high stability. Growing evidence has shown that circRNAs play critical roles in the development and progression of diseases, particularly in cancer growth, metastasis, stemness, and resistance to therapy.^10^ lncRNAs are a group of functional ncRNAs with a wide range of major regulatory functions in proliferation and differentiation, as well as tumor progression or tumor suppression.^6^^,^^11^, ^12^, ^13^, ^14^ Here, we review the current information on the role of ncRNAs in glioma, particularly their effects on cancer stem cells (CSCs).

### CSCs and glioma

**Figure 1:**
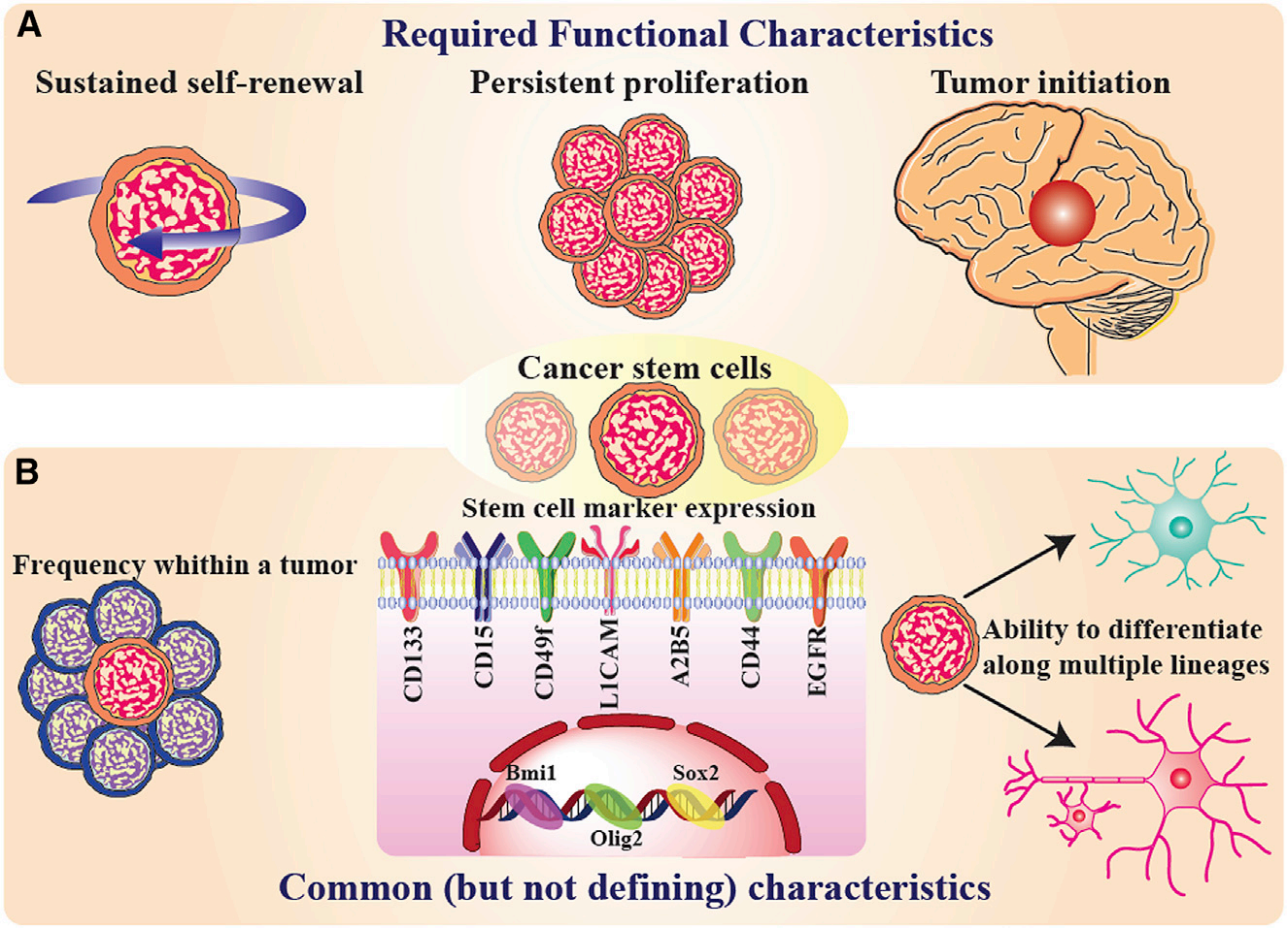
Functional criteria of CSCs (A) CSCs are defined by functional characteristics that include sustained self-renewal, persistent proliferation, and tumor initiation upon intracranial transplantation, which is the definitive functional CSC assay. (B) CSCs also share features with somatic stem cells, including frequency within a tissue or tumor, stem cell marker expression (examples relevant to GBM and the brain are provided), and the ability to generate progeny with multiple lineages. Bmi1, B cell-specific Moloney leukemia virus insertion site 1; Olig2, oligodendrocyte transcription factor 2; Sox2, SRY-box transcription factor 2. This figure was adapted from Lathia et al.^17^

The cellular heterogeneity in CNS tumors has long been appreciated^15^^,^^16^; however, the role of self-regenerating tumor cells with increased tumorigenesis has been poorly recognized. Up to now, different terms have been used to denote these cells, including tumor/cancer/brain stem cells, stem-like tumor cells, tumor/cancer/glioma/brain tumor-propagating cells, and glioma/cancer/brain tumor-initiating cells. Because of these inconsistencies, attention has shifted away from their biology and their role in tumorigenesis, toward the discovery of new markers expressed on these cells, and determining if these cells can replicate as floating (non-adherent) spheroids. Moreover, these tumor cells are not necessarily produced from transformed stem cells, and other cell types, including normal stem cells and well-differentiated progenitor cells, could undergo oncogenic transformation. Therefore, precise functional assays must be performed, and an accepted definition should be used in all experimental studies. Any population of CSCs must have the capacity for self-regeneration, and also be able to produce well-differentiated progeny (Figure 1). In the case of brain tumors, these cells can form a tumor following intracranial transplantation, recapitulating the heterogeneity of parental tumor cells. Tumor-initiating cells in animal models can be used for investigation, but CSCs are more infiltrative capability than their progeny, and also their progeny lose tumorigenic potential during differentiation. The presence of a cellular hierarchy can be demonstrated by prospective enrichment and depletion of tumorigenic and non-tumorigenic cells. Cancer cells that contain a cellular hierarchy and are tumorigenic, are considered glioma stem cells or glioma CSCs. Cell culture spheroids can be derived from brain cells (normal or neoplastic), and their progenitors have limited self-renewal potential. However, the mere ability to form spheroids does not define CSCs, without showing a self-renewing population.^18^ High-passage cell lines are unlikely to be functionally validated CSC models, and cannot accurately represent tumor complexity *in vivo*.^19^

At high passage numbers, cell lines exhibit changes in morphology, reduced or altered key functions and efficiency, and frequently no longer represent reliable models of their original source material due to selective pressures and genetic drift. Cancer cell lines have significant limitations due to a lack of vascular, stromal, and immune components. Tumors are ecosystems of evolving clones that compete or cooperate with each other and other normal cells that infiltrate their microenvironment.^20^ This begs the interesting question of whether these clones were selected during their growth into the culture medium or through cell passaging over time. As a result, cell lines derived from a single clone are not always representative of the diversity present in the original tumor.^21^

Thus, whereas the growth of glioma cells as neurospheres is not essential to retaining stemness, the microenvironment, including medium composition and culture conditions, influences the CSC properties.^18^^,^^22^^,^^23^

**Figure 2:**
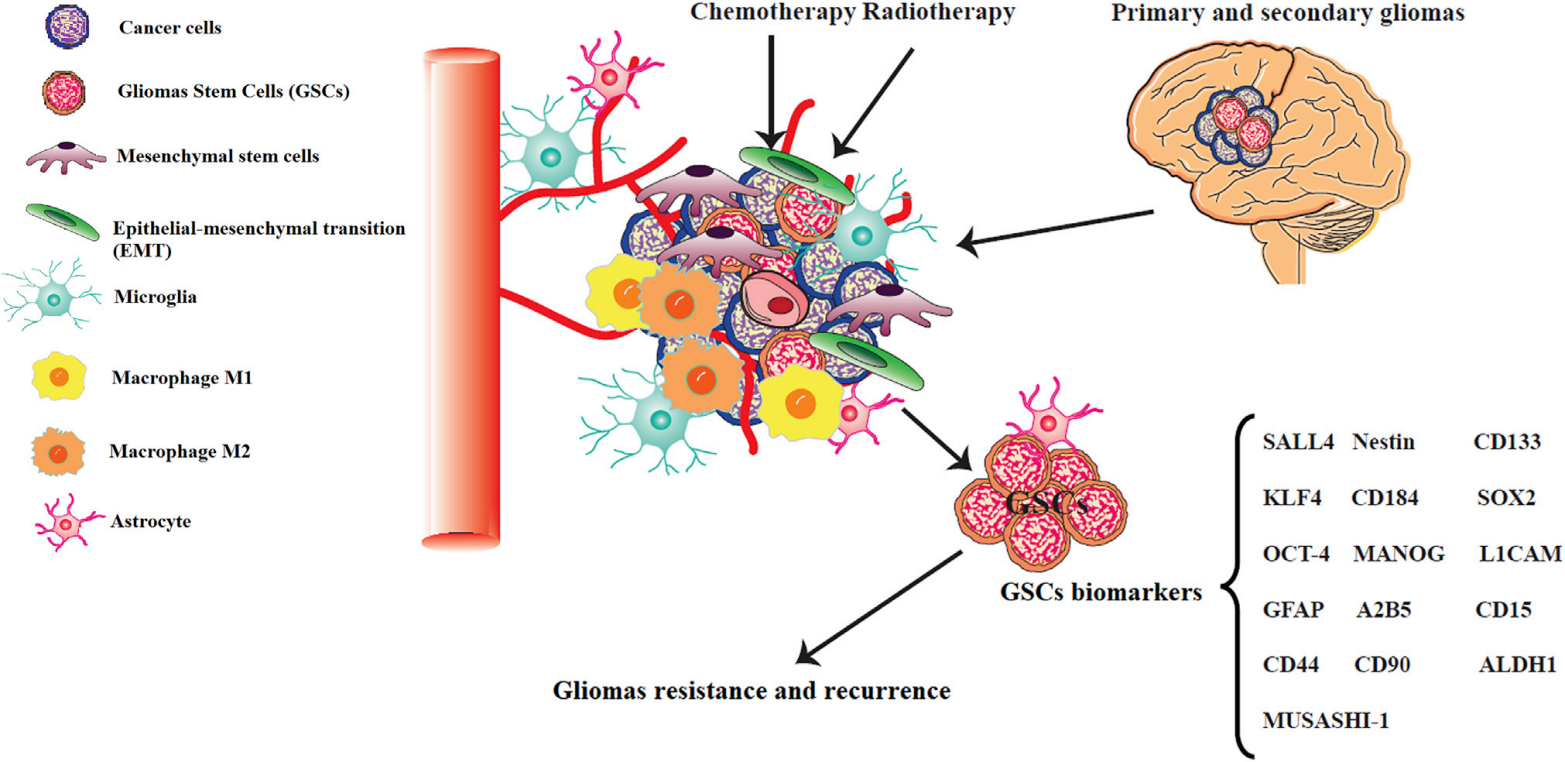
Schematic overview of the cellular components of the microenvironment of GBM The tumor microenvironment is a complex network composed of stromal cells (fibroblasts, microglia, astrocytes), mesenchymal cells, stem cells, and immune and inflammatory cells (macrophages). The main biomarkers of GSCs are indicated. This figure was adapted from Alves et al.^44^

After the adoption of CD133 as the first surface marker for GCSs, they were classified as CD133^+^ and CD133^−^. CD133^+^ cells or CSCs gradually lose their ability to self-renew during differentiation, but CD133 expression allows brain tumors to form *in vivo*, and neurospheres to grow *in vitro*.^22^^,^^24^, ^25^, ^26^, ^27^ Although other surface markers have been reported, which could be used to classify GSCs, the most useful marker remains CD133.^28^ Prognostic indicators for GBM progression, include CD133^+/^Ki-67^+^ cells, and the expression of HOX or Nestin genes.^29^, ^30^, ^31^ CD184 (CXCR4 chemokine-receptor) is another surface marker that is significantly correlated with CD133^+^ cells and has been shown to increase the expression of hypoxia-inducible factor 1 (HIF-1).^32^^,^^33^ Another surface marker is MUSASHI-1, which regulates the cell cycle and is an RNA binding protein involved in post-transcriptional gene editing.^34^ Many additional surface markers have been described that might be used to identify GSCs, such as the cell surface gangliosides GFAP, KLF4, SALL4, ALDH1, L1CAM, SOX2, CD90, and A2B5, and the cell surface glycoprotein CD44.^34^, ^35^, ^36^, ^37^, ^38^, ^39^, ^40^ Although CD133 is a cell surface marker that enriches GSCs, the use of CD133 as a unique glioma stem cell marker is likely not enough to tag the whole self-renewing cancer cell reservoir and additional research is needed to identify more markers for GBM stem cells.^41^, ^42^, ^43^ The main biomarkers of glioma stem cells are illustrated in Figure 2.

### miRNAs and CSCs in glioma

Members of the miR-17 family, miR-20a and miR-106a are expressed in multiple types of cells.^45^ Both upregulation and downregulation of miRNAs have been observed in various cancers, and specific miRNAs may either promote or suppress tumor formation. 46, 47, 48, 49, 50 miRNAs have also been implicated in the function of stem cells (both normal and cancer). For instance, upregulation of the miR-17-92 cluster (including miR-20a) induces pulmonary epithelium progenitor cells to proliferate and prevents them from differentiating.^51^ In mouse embryos, miR-20a/106a acts to control stem cell differentiation.^52^ miRNAs are also present in high levels in *MLL* leukemia stem cells and could affect their function by regulating p21.^53^ The anti-tumor activity of tissue inhibitor of metalloproteinases-2 (TIMP-2) has been reported in CSCs in various studies.^54^, ^55^, ^56^

miR-106a, one of the tumor-suppressor miRNAs, has played a significant role in the development and progression of human tumors. It was upregulated in colorectal cancer,^57^ gastric carcinoma,^58^ and mantle cell lymphoma,^59^ whereas it was downregulated in glioma. In their study, Dai et al. found that overexpression of miR-106a downregulated expression of glucose transporter 3 (GLUT3) or SLC2A3, an oncogene in several human cancers, via targeting 3′ UTR of SLC2A3, resulted in suppression of cell proliferation and cell glucose uptake in GBM cells.^60^ miR-20a is widely upregulated in diverse cancer subtypes, including hepatocellular cancer, lung cancer, and GBM.

The bioinformatic analysis confirmed using a luciferase reporter assay showed that miR-20a can directly and negatively regulate CELF2 (CUGBP Elav-like family member 2) gene expression, thus playing a critical role in the growth and invasion of glioma cells.^61^ In addition, miR-20a may regulate cell invasion of GBM via IL-6/JAK2/STAT3 axis belonging to the JAK/STAT signaling pathway.^62^ Xu et al. suggested that miR-20a introduces its oncogenic activity by the HIF-1a/c-MYC pathway in IDH1 R132H-mutant glioma.^63^ The presence of this mutation in glioma upregulates HIF-1a expression, which decreases c-MYC activity, resulting in a consequential decline in miR-20a, is responsible for glioma cell proliferation and resistance to temozolomide (TMZ) treatment.^63^

TIMP-2 is another target gene of miR-20a/106a, which can deregulate the expression of the TIMP-2 gene by interfering with the 3′ UTR of TIMP-2 mRNA in GBM.

Nordy (dl-nordihydroguaiaretic acid) is a small-molecule lipoxygenase inhibitor,^64^ which has been found to suppress cancer growth.^65^, ^66^, ^67^ *In vitro* as well as *in vivo* studies in glioma have shown its ability to modulate differentiation and inhibit growth.^66^ It was proposed that Nordy could drive GSCs toward differentiation,^65^ by increasing the expression of TIMP-2 via downregulation of miR-20a and miR-106a. Wang et al. examined the ability of miR-20a/106a to promote the invasion of GSCs.^45^ Compared with regular glioma cells, GSCs had higher expression of miR-20a/106a, which was associated with their invasion. miR-20a/106a was found to target TIMP-2 expression, which showed a negative correlation with the levels of these miRs. miR-20a/106a downregulation resulted in an increase in TIMP-2, which suppressed GSC invasion. The ability to suppress miR-20a/106a and therefore upregulate TIMP-2 was proposed to explain the anti-tumor effect of Nordy.^45^

Both anti-cancer and pro-oncogenic effects of miR-146a have been reported,^68^, ^69^, ^70^, ^71^, ^72^ and it has also been shown to be associated with prolonged survival in GBM patients.^73^

POU3F2 is a transcription factor belonging to the POU-domain transcription factor and is a key differentiation factor in neurons and embryonic development.^74^ POU3F2 knockdown can cause tumor-suppressor effects in various cancers.^75^, ^76^, ^77^, ^78^ It can be targeted to prevent prostate tumors from neuroendocrine differentiation.^78^ CircPOLR2A, an upregulated circRNA in GBM cells, activates the transcription of Sox9 through the miR-2113/POU3F2 axis, thus enhancing GBM cell growth.^79^ POU3F3 modulates cell proliferation by G1 cell-cycle arrest and apoptosis via its influence on DLL1 and Sox2. One study showed that long intergenic ncRNA POU3F3 (linc-POU3F3) is overexpressed in high-grade glioma tissues and promotes cell viability and proliferation of glioma cells, and leads to glioma progression through downregulation of POU3F3.^80^ In a recently published paper by Yang et al.,^81^ they reported that overexpression of POU2F2 significantly correlated with poor prognosis of GBM patients.^81^^,^^82^ They indicated that POU2F2 induces a metabolic shift toward aerobic glycolysis and promotes cell growth and GBM progression through PDPK1-dependent activation of the PI3K/AKT/mTOR pathway.^81^^,^^82^

SMARCA5 is a member of the SWI/SNF family, with helicase and ATPase properties. SMARCA5 can enhance cancer development in ovarian and glioma tumors.^83^^,^^84^ The suppressive effects of miR-100 on breast cancer stem cells could be partly mediated by regulating SMARCA5.^85^ Interestingly, the level of miR-146a was negatively associated with SMARCA5 in bladder cancer.^86^ Cui et al. investigated the role of miR-146a and its downstream pathways in GBM.^82^ They reported a significant downregulation of miR-146a as a result of promoter hypermethylation in recurrent GBM patients, which was associated with a poor prognosis. *In vitro* as well as *in vivo* findings showed that miR-146a upregulation greatly reduced proliferation and invasion, as well as the stemness of glioma cells, and enhanced their TMZ sensitivity. At the molecular level, these effects suggest the ability of miR-146a to suppress POU3F2 and SMARCA5 by directly targeting their 3′ UTRs in GBM cells. Altogether, their results suggest that miR-146a may inhibit stemness properties in GBM cells, and enhance their TMZ sensitivity.^82^

miR16 could suppress invasion and migration in glioma cells.^87^, ^88^, ^89^ In GBM cells, miR16 suppressed invasion, adhesion, and downregulated genes involved in epithelial-mesenchymal transition (EMT).^90^ Since an association between SOX2 and stemness has been reported, investigating the interplay between miR16 and the SOX family transcription factor in GSCs may reveal useful information. SOX family members SOXD and SOXE have been implicated in glioma formation.^91^ The transcription factor SOX5 acts to maintain the chromatin configuration and regulates gene expression in various developmental pathways. SOX5 was able to suppress proliferation in glioma cells.^92^^,^^93^ Tian et al. examined the levels of miR16 in GBM SGH44, U87, and U251 cells, and in GSCs, how it affected tumor progression, and its role as a possible prognostic marker.^94^ Both *in vitro* and *in vivo*, upregulation of miR16 suppressed tumor progression, while its inhibition was associated with tumor promotion. There was a positive correlation between miR16 levels and GSCs’ ability to differentiate, and a negative correlation with migration, invasion, and the ability to form colonies. Bcl2, CCND1, CCNE1, CDK6, and SOX5 were identified as direct targets of miR16, and all these factors were downregulated by miR16 in cells. Finally, they showed a correlation between miR16 levels and clinical outcomes in GBM patients and suggested that the anti-cancer effect of miR16 involved GSCs.^94^

miR-182 was first detected in murine neurosensory tissues.^95^, ^96^, ^97^ Although miR-182 levels are scarce in the fetal period, it becomes upregulated after birth, where it can induce the retinal progenitor cells to terminally differentiate, as well as maintain their mature form.^96^ It may also induce differentiation and the mesenchymal-to-epithelial transition by modulating SNAI2.^98^ c-Met has been reported to be upregulated in GBM,^99^, ^100^, ^101^ where it promotes tumor invasion and proliferation.^102^^,^^103^

Hypoxia-inducible factor 2α (HIF2A) is secreted in hypoxic conditions to enhance GSC survival and proliferation, and there was a negative correlation between HIF2A levels and glioma prognosis.^104^ Another pro-oncogenic factor, Bcl2-like12 (Bcl2L12), was reported to be upregulated in GBM.^105^^,^^106^ Kouri et al. examined the role of miR-182 in GBM, and whether it could be a prognostic marker.^107^ They identified that miR-182 is a tumor suppressor, which could inhibit Bcl2L12, c-Met, and HIF2A, and subsequently could prevent GSC growth and stemness, and possibly improve GBM treatment response. The same results were observed *in vivo*. They demonstrated an association between miR-182 and GBM prognosis and suggested that miR-182 could suppress GSCs by inhibiting Bcl2L12, c-Met, and HIF2A.^107^

miR-302-367 has been shown to play an important role in mesendoderm differentiation.^108^ In addition, miR-302-367 may also regulate stemness properties in stem cells of different origins.^109^^,^^110^ It was shown that stemness transcription factors, such as Oct4, Sox2, and Nanog, could regulate miR-302-367 in ESCs, as well as early development of murine cells.^109^^,^^111^ SDF1 can bind to CXCR4 to regulate various signaling pathways, such as PLC, PI3K/AKT, and MAPK, and to affect multiple cellular processes.^112^ CXCR4 is involved in proliferation and motility and could promote the aggressive phenotype of glioma, which explains its correlation with poor patient prognosis.^33^^,^^113^^,^^114^ In GSCs, the SHH-GLI-NANOG axis was shown to regulate proliferation and stemness.^115^, ^116^, ^117^, ^118^ Fareh et al. investigated the role of the miR-302-367 cluster in GSCs.^119^ They used serum to suppress stemness in GSCs, and observed upregulation of the expression of this cluster. They found that miR-302-367 upregulation could inhibit the stemness and tumorigenicity of GSCs by suppressing CXCR4 and disrupting the SHH-GLI-NANOG pathway. They concluded that the miR-302-367 cluster suppressed GSC stemness and tumorigenicity by inhibiting CXCR4 and interfering with the SHH-GLI-NANOG pathway.^119^

Table 1 lists some microRNAs that have been reported to be involved in CSCs, and GSCs in particular.

**Table 1:** Role of microRNAs in cancer stem cells

microRNA	Expression	Target	Model (*in vitro*, *in vivo*, human)	Ref.
miR-26a	↑	AP-2α	*in vitro*, *in vivo*	Huang et al.^120^
miR-93	↑ (higher upregulation in PN GSCs than in MES GSCs)	BECN1/Beclin 1, ATG5, ATG4B, and SQSTM1/p62	*in vitro*, *in vivo*, human	Huang et al.^121^
miR-3940-5p	↓	CUL7, NF-κB	*in vitro*	Xu et al.^122^
miR-9-5p	↑	NAP1L1, FREM2	*in vitro*	Zottel et al.^123^
miR-124-3p	↑	SPRY1, NAP1L1, VIM	*in vitro*	Zottel et al.^123^
miR-21-5p	↓	SPRY1	*in vitro*	Zottel et al.^123^
miR-138-5p	↓	VIM	*in vitro*	Zottel et al.^123^
miR-1-3p	↓	NCL	*in vitro*	Zottel et al.^123^
miR-30a	↓	NT5E/Akt signaling pathway	*in vitro*, *in vivo*	Peng et al.^124^
miR-150-5p	↓	Wnt/β-catenin pathway	*in vitro*, *in vivo*	Tian et al.^125^
miR-100-5p	↓	SMARCA5, SMRT, AKT, ERK, ErbB3, ErbB2, p21	*in vitro*, *in vivo*	Alrfaei et al.^126^
miR-146b-5p	↓	SMARCA5, TGF-β pathway	*in vitro*, *in vivo*	Wang et al.^127^
miR-18a	↑	RORA, TNF-α, NF-κB	*in vitro*, *in vivo*	Jiang et al.^128^
miR-145	↓	SOX2-Wnt/β-catenin pathway	*in vitro*, *in vivo*	Qian et al.^129^
miR-29a	↓	PDGFC, PDGFA	*in vitro*, *in vivo*	Yang et al.^130^
miR-320a	↓	*PHF6*, *MMP16*, *MCL1*	*in vitro*, *in vivo*	Kang et al.^131^
miR-4496	↓	*ABCB1*, *ABCG2*, *MGMT*, *PHF6*,*MMP16*, *MCL1*	*in vitro*, *in vivo*	Kang et al.^131^
miR-26a	↑	PTEN, PI3K/Akt	*in vitro*, *in vivo*	Wang et al.^132^
miR-504	↓	Grb10	*in vitro*, *in vivo*	Bier et al.^133^
miR-486-5p	↑	PTEN, FoxO1	*in vitro*, *in vivo*	Lopez-Bertoni et al.^134^
miR-1300	↓	ECT2	*in vitro*	Boissinot et al.^135^
miR-603	↓	IGF1, IGF1R	*in vitro*, *in vivo*	Ramakrishnan et al.^136^
miR-200b	↓	CD133/PI3K/Akt signaling axis	*in vitro*	Liu et al.^137^
miR-107	↓	Notch2, MMP-12	*in vitro*, *in vivo*	Yuan et al.^138^
miR-302-367	↓	CXCR4/SDF1, SHH, cyclin D, cyclin A, E2F1	*in vitro*, *in vivo*	Fareh et al.^139^
miR-370-3p	↓	NEAT1, HMGA2, HIF1A	*in vitro*, *in vivo*	Lulli et al.^140^
miR-141	↓	Jagged1	*in vitro*, *in vivo*	Gao et al.^141^
miR-7-5p	↓	Yin Yang 1	*in vitro*	Jia et al.^142^
miR-33a	↑	PDE8A→PKA UVRAG→NOTCH	*in vitro*, *in vivo*	Wang et al., 2014^143^
miR-203	↓	–	*in vitro*	Deng et al.^144^
miR-128	↓	BMI1, SUZ12	*in vitro*	Peruzzi et al.^145^
miR-145	↓	ABCG2	*in vitro*	Shi et al.^146^
miR-148a	↑	GADD45A	*in vitro*, *in vivo*	Cui et al.^147^
miR-30	↑	Jak/STAT3, SOCS3	*in vitro*, *in vivo*	Che et al.^148^
miR-205	↓	–	*in vitro*, *in vivo*	Huynh et al.^149^
miR-300	↑	LZTS2	*in vitro*, *in vivo*	Zhang et al.^150^
miR-143	↓	hexokinase 2	*in vitro*, *in vivo*	Zhao et al.^151^
miR-10b	↑	–	*in vitro*, *in vivo*	Guessous et al.^152^
miR-146b-5p	↓	HuR/lincRNA-p21/β-catenin axis	*in vitro*, *in vivo*	Yang et al.^153^
miR-124	↓	KITL, SEMA6D, NRP2, THBS1	*in vitro*, *in vivo*	Marisetty et al.^154^
miR-203	↓	KITL, SEMA6D, NRP2, THBS1	*in vitro*, *in vivo*	Marisetty et al.^154^
miR-34a	↓	cyclin D1, c-myc, c-met, Ki-67 Bcl-2 Family	*in vitro*	Sun et al.^155^
miR-30b-3p	↑	RHOB	*in vitro*, *in vivo*	Yin et al.^156^
miR-135a	↓	Arhgef6	*in vitro*, *in vivo*	Hemmesi et al.^157^
miR-138	↑	CASP3, BLCAP, MXD1	*in vitro*, *in vivo*	Chan et al.^158^
miR-153	↓	Dvl-3	*in vitro*	Zhao et al., ^159^
miR-146a	↑	NUMB	*in vitro*	Puca et al., ^160^
miR-608	↓	MIF	*in vitro*	Wang et al., ^161^
miR-10b	↑	P21, P16, BIM, PTBP2	*in vitro*, *in vivo*	El Fatimy et al., ^162^
miR340	↓	PLAT	*in vitro*, *in vivo*	Yamashita et al., ^163^
miR-34a	↓	c-Met, Notch	*in vitro*	Guessous et al.^91^
miR-20a/106a	↑	TIMP-2	*in vitro*, *in vivo*	Wang et al., ^45^
miR-21	↑	FASLG	*in vitro*	Shang et al., ^80^
miR-135b	↓	ADAM12, SMAD5, GSK3β	*in vitro*, *in vivo*	Lulli et al., ^164^
miR-223	↑	PAX6, PI3K/Akt	*in vitro*	Huang et al., ^165^
miR-153	↓	Nrf-2/GPx1/ROS axis	*in vitro*, *in vivo*	Yang et al., ^166^
miR-125b	↓	E2F2	*in vitro*	Wu et al., ^167^
miR-146a	↓	POU3F2, SMARCA5	*in vitro*, *in vivo*, human	Cui et al., ^82^
miR-451	↓	–	*in vitro*	Gal et al., ^168^
miR-124	↓	STAT3	*in vitro*, *in vivo*	Wei et al., ^169^
miR-134b	↓	MMP-12	*in vitro*, *in vivo*	Liu et al., ^170^
miR-218	↓	Bmi1, Wnt	*in vitro*, *in vivo*	Tu et al., ^171^
miR-23b	↓	HMGA2	*in vitro*	Geng et al., ^172^
miR-296-5p	↓	HMGA1, Sox2	*in vitro*, *in vivo*	Lopez-Bertoni et al., ^173^
miR-125b-2	↑	Bax, Bcl-2, cytochrome c, Apaf-1, caspase-3, PARP	*in vitro*	Shi et al., ^174^
miR-198	↑	NNAT	*in vitro*	Liu et al., ^175^
miRNA-155-5p	↑	BMP	*in vitro*	Liu et al., ^175^
miRNA-124-3p	↓	–	*in vitro*	Liu et al., ^175^
miR-455-3p	↑	Smad2	*in vitro*, human	Tezcan et al., ^176^
miR-181b	↓	–	*in vitro*, human	Tezcan et al., ^176^
miR-125b	↑	Bak1	*in vitro*, *in vivo*	Chen et al., ^177^
miR-137	↓	RTVP-1	*in vitro*	Bier et al., ^178^
Let-7b	↓	E2F2	*in vitro*	Song et al., ^179^
miR-145	↓	CTGF, SPARC	*in vitro*, human	Lee et al., ^180^
miR-92a-3p	↑	CDH1/β-catenin, Notch1/Akt	*in vitro*, *in vivo*	Song et al., ^181^
miR128-1	↓	BMI1, E2F3	*in vitro*, *in vivo*	Shan et al., ^182^
miR-148a	↑	MIG6, BIM	in *vitro*, *in vivo*, human	Kim et al., ^183^
miR-125b	↑	MMP9	*in vitro*, *in vivo*	Wan et al., ^184^
miR-181b	↓	–	*in vitro*	Li et al., ^185^
miR16	↓	Bcl2, CDK6, CCND1, CCNE1, SOX5	*in vitro*, *in vivo*	Tian et al., ^94^
miR-182	↓	Bcl2L12, c-Met, HIF2A	*in vitro*, *in vivo*, human	Kouri et al., ^107^
miR-152	↓	KLF4, LGALS3, MEK1/2, PI3K	*in vitro*, *in vivo*	Ma et al., ^37^
miR-29a	↓	QKI-6/WTAP	*in vitro*, *in vivo*	Xi et al., ^186^
miR-125b	↓	CDK6, CDC25A	*in vitro*	Shi et al., ^187^
miR-449a	↑/↓	Ccnd1, Gpr158	*in vitro*, *in vivo*, human	Li et al., ^188^
miR-7	↓	EGFR, Akt, NF-κB, DR5	*in vitro*, *in vivo*	Bhere et al., ^189^
miR-9/9^∗^	↑	CAMTA1, NPPA	*in vitro*, *in vivo*, human	Schraivogel et al., ^190^
miR-18a∗	↑	ERK, DLL3, NOTCH, SHH-GLI-NANOG axis	*in vitro*, *in vivo*	Turchi et al., ^191^
miR-145	↓	NEDD9	*in vitro*, *in vivo*, human	Speranza et al., ^192^
miR-128	↓	Bmi-1	*in vitro*, *in vivo*	Godlewski et al., ^193^
miR-145	↓	JAM-A	*in vitro*, human	Alvarado et al., ^194^
miR-126	↑	–	*in vitro*, *in vivo*	Halle et al., ^195^
miR-137	↑	–	*in vitro*, *in vivo*	Halle et al., ^195^
miR-128	↑	–	*in vitro*, *in vivo*	Halle et al., ^195^
miR-218-5p	↓	MMP9	*in vitro*	Wu et al., ^196^
miR-128	↓	EGFR, PDGFRα	*in vitro*, *in vivo*	Papagiannakopoulos et al., ^197^
miR-374a	↓	NRN1, CCND1, CDK4, Ki67	*in vitro*, *in vivo*	Pan et al., ^198^
miR-125b	↓	Lin28	*in vitro*	Wan et al., ^199^
miR-491	↓	IGFBP2, EGFR, CDK6, Bcl-xL	*in vitro*, *in vivo*	Li et al., ^200^
miR-101	↓	KLF6, CHI3L1, MEK1/2, PI3K	*in vitro*, *in vivo*	Yao et al., ^201^
miR-139	↓	PDE2A, FZD3, β-catenin, GSK-3β	*in vitro*, *in vivo*, human	Li et al., ^202^
miR-154	↑	PRPS1	*in vitro*, *in vivo*	Yang et al., ^203^
miR-124	↓	CDK6, pSer 807/811	*in vitro*	Silber et al., ^204^
miR-137	↓	CDK6, pSer 807/811	*in vitro*	Silber et al., ^204^
miR-330	↑	SH3GL2, ERK, PI3K/AKT	*in vitro*, *in vivo*	Yao et al., ^205^
miR-302-367 cluster	↓	CXCR4/SDF1, SHH/GLI/NANOG	*in vitro*, *in vivo*	Fareh et al., ^119^
miR-363	↑	caspase-3, caspase-9, Bim	*in vitro*	Floyd et al., ^206^
miR-582-5p	↑	caspase-3, caspase-9, Bim	*in vitro*	Floyd et al., ^206^
miR-145	↓	Sox9, ADD3	*in vitro*, *in vivo*	Rani et al., ^207^
miR-10	↑	CSMD1, HOXD10	*in vitro*	Lang et al., ^208^
miR-124	↓	NRAS, PIM3	*in vitro*	Lang et al., ^208^
miR-125b	↑	PIAS3	*in vitro*, *in vivo*	Shi et al., ^209^
miR-134	↓	KRAS, STAT5B	*in vitro*, *in vivo*	Zhang et al., ^210^
miR142-3p	↓	IL-6, HMGA2, Sox2	*in vitro*, *in vivo*, human	Chiou et al., ^211^
CMV70-3P	↑	SOX2	*in vitro*	Ulasov et al., ^212^
miR-148a	↓	–	*in vitro*, *in vivo*	Lopez-Bertoni et al., ^213^
miR-124	↓	PTBP1, ANXA7	*in vitro*	Ferrarese et al., ^214^
mir34c	↓	Bcl2, NUMB, p73, AKT	*in vitro*	Iannolo et al., ^215^
miR-944	↓	VEGFC, AKT/ERK	*in vitro*, *in vivo*	Jiang et al., ^216^
miR-34a	↓	Rictor, PI3K/AKT, WNT/β-Catenin	*in vitro*, *in vivo*	Rathod et al., ^217^
miR-425-5p	↑	FOXJ3, RAB31	*in vitro*, *in vivo*	De La Rocha et al., ^218^
miR-451	↓	AMPK/OCT1/miR-451/LKB1	*in vitro*, *in vivo*	Ogawa et al., ^219^
miR-24-3p	↑	BNIP3	*in vitro*, *in vivo*	Zhang et al., ^220^

### lncRNAs and CSCs in glioma

**Figure 3:**
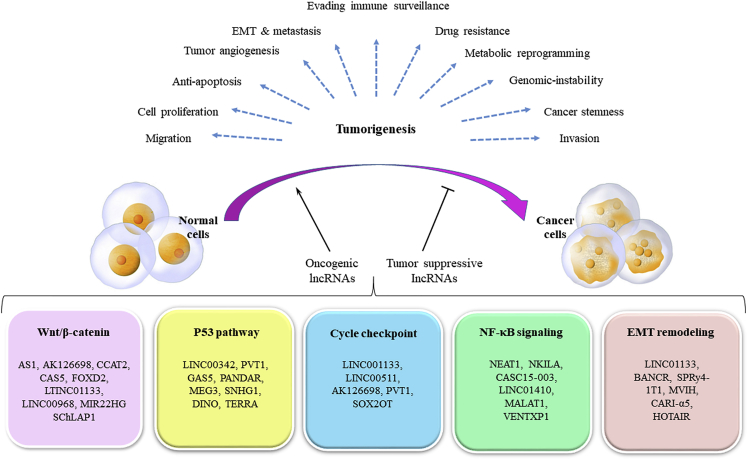
The role of lncRNAs in regulating cancer cellular processes Roles of oncogenic versus tumor suppressive lncRNAs and their mechanisms in tumorigenesis.

lncRNAs, through several mechanisms, are involved in metabolic reprogramming, cell proliferation, cell apoptosis, cell metastasis and invasion, cell-cycle and genomic instability, EMT and migration, cancer stemness, and drug resistance (Figure 3).^221^ The oncogenic function of the lncRNA NEAT1 has been shown in glioma and other tumors.^222^, ^223^, ^224^ Low levels of Let-7g-5p (a let-7 family member) have been reported in glioma patient samples,^225^ and higher levels may be predictive of better clinical outcomes in GBM.^226^

MAP3K1 has pro-oncogenic effects in glioma, gastric, and breast cancer by regulating proliferation and migration, as well as promoting tolerance to therapy.^227^, ^228^, ^229^ Bi et al. investigated the level and function of NEAT1 in GSCs.^230^ They observed higher levels of NEAT1 in GSCs, as well as in the serum of GBM patients. Suppression of NEAT1 was able to prevent GSCs from proliferating, migrating, and invading. Similar results were found after upregulation of let-7g-5p, which was identified as a direct target of NEAT1. Next, they found that let-7g-5p exerted its effects by targeting and inhibiting MAP3K1. Taken together, their data suggested that NEAT1 could promote GSC pro-oncogenic activity and TMZ tolerance by regulating the let-7g-5p/MAP3K1 pathway.^230^

Esophageal squamous cell cancer and glioma have both been reported to show increased levels of MALAT1.^231^^,^^232^ miR-129-3p, miR-129-2-3p, and miR-129–5p are three important members of the miR-129 family.^233^ miR-129–5p has tumor-suppressor roles in various cancers, such as ovarian,^234^ breast,^235^ and glioma.^236^ In glioma, miR-129 overexpression showed an anti-oncogenic effect by regulating the Notch-1/E2F7/Beclin-1 pathway.^237^ SOX2 is regarded as a molecular signature of GSCs, as well as pluripotent stem cells.^238^^,^^239^ Xiong et al. examined the role of MALAT1 in GSCs and the interactions between this lncRNA with miR-129 and SOX2.^240^ Compared with regular (non-stem) glioma cells, GSCs showed higher levels of MALAT1, but lower levels of miR-129. MALAT1 inhibition could impair GSC proliferation by upregulating miR-129. miR-129 was found to target and inhibit SOX2. These effects were also observed *in vivo*. They concluded that MALAT1 could promote GSC tumorigenicity both *in vitro* and *in vivo*, by regulating the miR-129/ SOX2 axis.^240^

lncRNA TP73-AS1 was found to be epigenetically downregulated in both oligodendroglioma and GBM.^241^^,^^242^ In addition, GBM patient prognosis was found to be positively correlated with TP73-AS1 levels.^243^ ALDH1A1 has been identified as a marker of GSCs,^244^^,^^245^ and is involved in GSC progression and therapy resistance.^245^, ^246^, ^247^ It was proposed that ALDH1A1 could interfere with the oxidative stress triggered by chemotherapeutic drugs and induce tolerance to treatment.^246^ Mazor et al. studied the effects of TP73-AS1 in GBM patients and GSCs.^248^ High levels of TP73-AS1 were observed in GBM patients and were correlated with poor clinical outcomes. They found that TP73-AS1 could attenuate the response of GSCs to TMZ treatment, which could be attributed to its ability to regulate ALDH1A1. Finally, they found a correlation between TP73-AS1 overexpression and poor clinical outcomes in GBM patients. They suggested that TP73-AS1 could enhance tumorigenicity in GSCs and reduce their sensitivity to TMZ by upregulating ALDH1A1.^248^

XIST (X-inactive-specific transcript) is a lncRNA gene on the X chromosome of placental mammals, which produces a lncRNA to silence one of the paired X chromosomes in females.^249^ Aberrant XIST expression has been observed in several cancers, and its oncogenic effect may be explained by causing instability in the heterochromatin structure.^250^ Furthermore, lncRNA XIST may promote the viability of hematopoietic stem cells.^251^ Yao et al. investigated the role of XIST in human GSCs.^250^ They found that both glioma cells and GSCs had elevated levels of XIST. *In vitro* downregulation of XIST in GSCs reduced proliferation, migration, and invasion, while promoting apoptosis and suppressing oncogenesis. The same results were observed after XIST downregulation in a murine model. They identified miR-152 as a direct target of XIST to explain its function. miR-152 has been shown to exert anti-oncogenic effects in GSCs by regulating KLF4.^37^ In conclusion, they identified XIST as an oncogene whose suppression could reduce the oncogenesis of GSCs by upregulating miR-152.^252^

Fibroblasts are a group of stromal cells in the tumor microenvironment (TME), which may promote tumor cell progression and metastasis.^253^^,^^254^ Cancer cells can progressively activate normal fibroblasts within their environment to form cancer-associated fibroblasts (CAFs).^255^ Because of the importance attributed to CAFs in the TME, investigating their potential as targets to treat gliomas is of increasing interest.^256^ There is an association between the CAF abundance in the TME and poor clinical outcomes.^257^^,^^258^ lncRNA HOTAIRM1 shares the same location as HOX genes and has been reported to have pro-oncogenic or anti-oncogenic effects in various cancers by regulating HOXA genes.^259^, ^260^, ^261^, ^262^, ^263^ The anti-oncogenic effects of miR-133b have been proposed to be mediated by different molecules in different cancers, including HOTAIRM1.^264^, ^265^, ^266^, ^267^, ^268^ Wang et al. explored the interaction between GSCs and fibroblasts in TME, both *in vitro* and *in vivo*, and the underlying molecular pathways.^269^ GSCs were able to trigger fibroblasts to behave as malignantly transformed fibroblasts (t-FBs). They observed elevated levels of HOTAIRM1 in both glioma cells and t-FBs. In addition, a correlation between high HOTAIRM1 levels and poor clinical outcomes was observed in glioma patients. HOTAIRM1 knockdown suppressed the pro-tumorigenic and malignant behavior of t-FBs, while the opposite effect was observed by HOTAIRM1 upregulation. At the molecular level, HOTAIRM1 was found to target and inhibit miR-133b-3p, which in turn upregulated TGF-β. Taken together, HOTAIRM1 was identified as an oncogene in GSCs, which could modulate the t-FB malignant behavior by inhibiting miR-133b-3p and upregulating TGF-β.^269^

Table 2 lists some lncRNAs that have been reported to be involved in CSCs, and GSCs in particular.

**Table 2:** Role of lncRNAs in cancer stem cells

LncRNA	Expression	Target	Model (*in vitro*, *in vivo*, human)	Ref.
TUG1	↑	Nestin, miR-145, SOX2, MYC, PRC2 components (EZH2, SUZ12), YY1, BDNF, NGF, NTF3	*in vitro*, *in vivo*	Katsushima et al.^270^
LINC00115	↑	miR-200s, ZEB1, ZNF596/EZH2/STAT3 signaling pathway	*in vitro*	Tang et al.^271^
MALAT1	↑	miR-129-5p, HMGB1	*in vitro*	Yang et al.^272^
Linc00152	↑	miR-103a-3p/FEZF1/CDC25A axis	*in vitro*, *in vivo*	Yu et al.^273^
GAS5	↓	miR-196a-5p/FOXO1/PID1, MIIP pathway	*in vitro*, *in vivo*	Zhao et al.^274^
HOTAIRM1	↑	HOX genes	*in vitro*, *in vivo*	Xia et al.^275^
PCAT1	↑	miR-129–5p, HMGB1	*in vitro*	Zhang et al.^276^
NEAT1	↑	let-7g-5p/MAP3K1	*in vitro*, human	Bi et al.^230^
MALAT1	↑	miR-129, SOX2	*in vitro*, *in vivo*	Xiong et al.^240^
NEAT1	↑	let-7e, NRAS	*in vitro*, *in vivo*	Gong et al.^222^
TP73-AS1	↑	ALDH1A1	*in vitro*, human	Mazor et al.^248^
CRNDE	↑	miR-186/XIAP, PAK7	*in vitro*, *in vivo*	Zheng et al.^277^
TALNEC2	↑	miR-21, miR-191	*in vitro*, *in vivo*	Brodie et al.^278^
NEAT1	↑	miR-107, CDK6	*in vitro*	Yang et al.^279^
XIST	↑	miR-152	*in vitro*, *in vivo*	Yao et al.^252^
SNHG9	↑	miR-326/SOX9	*in vitro*	Wang et al.^280^
Linc01060	↑	MZF1/c-Myc/HIF1α	*in vitro*, *in vivo*, human	Li et al.^281^
MIR22HG	↑	miR-22-3p, miR-22-5p, SFRP2, PCDH15, Wnt/β-catenin	*in vitro*, *in vivo*, human	Han et al.^282^
ASB16-AS1	↑	E-cadherin, N-cadherin, vimentin, EMT	*in vitro*, human	Zhang et al.^283^
lncRNA-ZNF281	↓	NF-κB1	*in vitro*, *in vivo*	Li et al.^284^
MALAT1	↑	MRP1, Bcl-2, HSP70, IAPs, p53	*in vitro*, *in vivo*	Kim et al.^285^
ENSG00000235427.1	↓	CAV1	*in vitro*	Li et al.^286^
ENSG00000261924.1	↓	RPTOR	*in vitro*	Li et al.^286^
P2RX5-TAX1BP3	↓	TAX1BP3	*in vitro*	Li et al.^286^
MALAT1	↑	ERK/MAPK	*in vitro*	Han et al.^287^
lincRNA-ROR	↓	KLF4	*in vitro*	Feng et al.^288^
H19	↑	–	*in vitro*	Li et al.^289^
HIF1A-AS2	↑	IGF2BP2, DHX9, HMGA1	*in vitro*, *in vivo*	Mineo et al.^290^
TUG1	↓	EZH2	*in vitro*, *in vivo*	Cao et al.^291^
HOXB-AS1	↑	–	*in vitro*	Shao et al.^292^
H19	↑	–	*in vitro*, *in vivo*, human	Jiang et al.^293^
SOX2OT	↑	miR-194-5p, miR-122, SOX3, TDGF-1	*in vitro*, *in vivo*	Su et al.^294^
RP11-279C4.1	↑	miR-1273g-3p/CBX3	*in vitro*, *in vivo*	Wang et al.^295^
HOTAIR	↑	EZH2, LSD1, PDCD4, CCND1, CDK4	*in vitro*, *in vivo*	Fang et al.^296^
MEG3	↓	vimentin, β-actin, Src_pY527, FAK_pY397, caveolin-1, connexin-43, NDRG1_pT346	*in vitro*, *in vivo*, human	Buccarelli et al.^297^
HOTAIRM1	↑	miR-133b-3p/TGF-β	*in vitro*, *in vivo*, human	Wang et al.^269^

### Enhancer RNAs

Enhancer RNAs (eRNAs), a new subclass of lncRNAs, participate in the regulation process of gene transcription.^298^ A growing number of studies showed that eRNAs interact with transcription factors, RNA-binding proteins, and transcriptional coactivators, such as CBP/p300 and Bromodomain-containing protein 4.^299^ Another mechanism discovered to underlie eRNA functions is that eRNAs participate in transcription factor trapping to increase their local concentration at DNA at the site of transcription.^299^^,^^300^

Based on the evidence, eRNAs play a critical role not only in cell development and homeostasis but also indirectly drive human diseases and differentiation. The most recent findings provide new insights into the characteristics and mechanisms of action of eRNAs, highlighting potentially broad roles of eRNA interactions in tumorigenesis and various cancer types.^301^^,^^302^ By modifying gene transcription and protein-RNA interactions, they can influence the expression of oncogenes and tumor-suppressor genes, as well as in abnormal cellular responses to external signals, such as inflammation, hypoxia, hormones, and other stimuli.^300^^,^^303^ Emerging studies also indicated the role of eRNAs in the regulation of key immune checkpoints and immune escape of tumor cells.^301^^,^^302^ For example, CCAT1, a super-enhancer-derived eRNA, induces PD-L1 expression via activating PI3K/AKT and RAS/MAPK pathways.^300^

Many eRNAs were found to be significantly overexpressed in tumor samples when compared with adjacent normal tissues. Because of their cancer-specific pattern of expression, eRNAs are clinically relevant and can serve as diagnostic, prognostic, and treatment response biomarkers in cancer therapy.^304^^,^^305^ In this setting, thanks to the efforts of scientists who attempted to infer cancer-specific expression of eRNAs from RNA sequencing data collected in numerous cancer series around the world,^306^^,^^307^ a systematic mapping of eRNAs expressed in various types of cancer is now available. The expression profiles of those eRNAs may help in eliminating intratumor heterogeneity and improving the diagnosis and treatment of a variety of cancers. For instance, focally amplified lncRNA on chromosome 1 (FAL1) has been recognized as an oncogene in numerous cancers and its overexpression is usually associated with poor prognosis.^308^ It supports cell proliferation and facilitates EMT, migration, and invasion by modulating the PTEN/AKT pathway. In addition, FAL1 contributes to the growth and metastatic potential of cancer cells via STAT3 phosphorylation and phosphorylation of GSK-3β, a protein crucial in Wnt signaling pathway regulation.^308^

Inflammatory signals have been shown to activate extensive programs of enhancer activation and eRNA production. Rahnamoun et al. revealed, in cancer cells, that p53 mutants abnormally activated a group of enhancers in response to pro-inflammatory TNF-α signaling.^309^ Co-binding of mutant p53 and NF-κB at these enhancers induced eRNA synthesis, one of which was necessary for the activation of key inflammation genes, such as C-C motif chemokine ligand 2 (CCL2).^310^ As a result, eRNAs play a direct role in cancer cell immune response. Many other cancer-related signaling pathways, including the Wnt, Notch, and Hippo pathways, orchestrate nuclear events, such as chromatin remodeling and transcription factor/cofactor recruitment to function by enhancer control.^303^

Lin et al. recently used the PreSTIGE computational pipeline to predict tissue-specific enhancer-derived RNAs and the underlying regulatory genes.^311^ They chose three eRNAs for their significant prognostic values to construct a risk signature: CRNDE, LINC00844, and MRPS31P5. Pathway and gene ontology analyses revealed that the risk signature in glioma is associated with mRNA processing and spliceosome. Furthermore, they discovered that hub eRNAs may regulate the expression of a variety of splicing factors, including MOV10 and SEC31B, and are associated with prognosis-associated alteration splicing. The researchers developed a risk signature composed of three eRNAs that can be used as targets to accurately predict prognosis in glioma patients.^311^ In another study, Guo et al., by functional enrichment analysis and immunogenomic profiling, indicated that AC003092.1 as an immune-related eRNA is related to glioma-immunosuppressive microenvironment.^312^

### circRNAs and CSCs in glioma

The circRNA Serpine2 is able to regulate the migration and invasion of glioma cells by modulating the expression of uPA and MMP-9/2.^313^ It was also able to promote the transformation of preneoplastic lesions into medulloblastoma.^314^ The tumor-suppressor role of miR-124-3p has been reported in glioma and GSCs.^175^^,^^315^ KIF20A was found to be a hub gene with an important function in p53 regulation,^316^ and was also highly elevated during mitotic processes in glioma cells.^317^ Using *in silico* prediction, Li and Lan tried to identify circRNA/miRNA/mRNA axes in GSCs,^318^ resulting in the identification of the Serpine2/miR-124-3p/KIF20A axis. Serpine2 was found to be upregulated in GSCs and could be delivered to glioma cells inside exosomes, to enhance glioma cell tumorigenesis. Serpine2 exerted its effects by inhibiting miR-124-3p and upregulating KIF20A. All these results were replicated *in vivo*. Serpine2 was identified as an oncogenic agent in GSCs, which could be transported to glioma cells to enhance their tumorigenicity by regulating the miR-124-3p/KIF20A axis.^318^

EGFR is reported to be highly expressed in about 50% of GBM tumors and has been recognized as an oncogene in GBM.^319^^,^^320^ Many studies have attempted to target EGFR to treat GBM, but the results have not so far been very successful.^321^, ^322^, ^323^ It has been found that circRNAs are readily translated^324^ because they lack a stop codon in their structure.^325^ Gao et al. investigated EGFR activity in GBM^326^ and discovered an additional pathway for activating EGFR independent of EGF. In this pathway, C-E-Cad (a variant of E-cadherin) was found to act as a ligand for EGFR. C-E-Cad is translated from circ-E-Cad, a translatable circRNA with high expression levels in GSCs, which enhances their tumorigenicity. Moreover, the efficacy of anti-EGFR therapy was significantly increased by suppressing C-E-Cad expression. In conclusion, they identified C-E-Cad as an independent activating ligand for EGFR in GBM, which could be targeted to improve the efficacy of anti-EGFR therapy.^326^

The Hedgehog (HH) signaling pathway is activated in various cancers and plays an important role in embryonic stem cells while it is silent in mature cells.^327^, ^328^, ^329^ The HH network includes HH ligands (Shh, Ihh, and Dhh), as well as PTCH, SMO, and Gli proteins.^330^ The HH pathway works as follows: first HH binds to PTCH to derepress SMO, then SMO prevents SUFU from inhibiting Gli1, and then the activated transcription factor Gli1 can regulate gene expression. Direct inhibition of SMO via PTCH has not yet been proven, but it has been found that cholesterol is needed to prevent PTCH from inhibiting SMO. In addition, cholesterol can endogenously activate SMO.^331^ Nevertheless, the exact mechanism for PTCH suppression of SMO is elusive, and understanding this step could clarify the whole HH pathway.^332^ Wu et al. explored the details of the HH pathway in GBM.^333^ They discovered a new protein called SMO-193a.a, which affects the HH pathway. SMO-193a.a is translated from circ-SMO (a translatable circRNA) in GSCs. Knockdown of SMO-193a.a disrupted the HH pathway in GSCs and reduced their tumorigenic ability both *in vitro* and *in vivo*. In addition, Gli1 could target FUS to upregulate SMO-193a.a, and the HH pathway activity is maintained in GSCs via the Shh/Gli1/FUS/SMO-193a.a axis. Clinically speaking, SMO-193a.a protein expression is more specific for GBM than SMO RNA expression and is better correlated with Gli1 levels. Furthermore, they also observed a correlation between SMO-193a.a levels and a poor prognosis in GBM patients. They concluded that SMO-193a.a could be translated from circSMO to increase the oncogenic capacity of GSCs via induction of the HH pathway.^333^

Recently, significant overexpression of circSCAF11 was discovered in glioma tissues and cell lines, and ectopic upregulation of circSCAF11 was found to be closely related to glioma patients' poor clinical outcome.^334^

Table 3 lists some circRNAs that have been reported to be involved in CSCs, and GSCs in particular.

**Table 3:** Role of circRNAs in cancer stem cells

Circular RNAs	Expression	Target	Model (*in vitro*, *in vivo*, human)	Ref.
circPTN	↑	miR-145-5p/miR-330–5p	*in vitro*, *in vivo*	Chen et al.^10^
Serpine2	↑	miR-124-3p/KIF20A	*in vitro*, *in vivo*	Li and Lan^318^
circCHAF1A	↑	FMR1/circCHAF1A/miR-211-5p/HOXC8, MDM2, p53	*in vitro*, *in vivo*, human	Jiang et al.^335^
cMELK	↑	miR-593/EphB2	*in vitro*, *in vivo*	Zhou et al.^336^
circATP5B	↑	miR-185-5p/HOXB5, JAK2/STAT3	*in vitro*, *in vivo*, human	Zhao et al.^337^
circ-E-Cad (translatable)	↑	EGFR-STAT3	*in vitro*, *in vivo*, human	Gao et al.^326^
circ-SMO (translatable)	↑	SMO	*in vitro*, *in vivo*	Wu et al.^333^
cARF1	↑	miR-342-3p/ISL2	*in vitro*, *in vivo*, human	Jiang et al.^338^

### Conclusions

The properties of stem cells are self-regeneration and differentiation into several lineages of normal cells, but CSCs may be caused by disturbance of these properties. The presence of CSCs in a tumor causes metastasis to spread more readily. In the brain, the rate and developmental timing of neurogenesis can be changed by the differentiation and self-renewal of cortical progenitor cells. The main reason for the development of glioma is a failure of cellular differentiation, but there is also evidence that aberrant epigenetic mechanisms involving ncRNAs are involved in glioma development. NcRNAs can regulate cellular signaling in CSCs and glioma cells. However, more research into the exact pathways and mechanisms of action of ncRNAs in CSCs and glioma is required to develop a more effective therapy for glioma patients. Recently, new studies have revealed the role of lncRNAs in embryonic pluripotency and self-renewal potential, but there is still a need for more studies into the exact role of ncRNAs in the transformation process, CSC therapy resistance, and maintaining stemness. Thus, ncRNAs could allow us to eventually achieve more success in glioma treatment. These in-depth studies of ncRNA biology will ultimately yield further insight into the molecular mechanisms of tumorigenesis, and lead to the development of improved therapeutic strategies against glioma, which are urgently needed. A summary of the function of glioma stem cell ncRNAs and its mechanism is given in Table 4.

**Table 4:** The summary of tumor stem cells ncRNAs role in glioma

ncRNA	Type	Effect	Mechanism	Ref.
FOXD2-AS1	lncRNA	promoting stemness and proliferation	recruiting TAF-1 to the NOTCH1 promoter region	Wang et al.^339^
circ-Serpine2	lncRNA	promoting proliferation, migration, and invasion	circ-Serpine2 could upregulate KIF20A by sponging miR-124-3p	Li and Lan^318^
RBM5-AS1	lncRNA	promotes radioresistance in medulloblastoma	stabilization of SIRT6 protein	Zhu et al.^340^
TUG1	lncRNA	alleviated TMZ resistance and inhibited tumorigenicity	downregulating EZH2 expression	Cao et al.^291^
SNHG9	lncRNA	facilitates growth of GSCs	competitive endogenous RNA of miR-326 to elevate the expression of SOX9	Wang et al.^341^
RP11-279C4.1	lncRNA	functions as an oncogene that promotes tumour progression	modulating the miR-1273g-3p/CBX3 axis	Wang et al.^295^
TPTEP1	lncRNA	inhibits stemness and radioresistance	miR-106a-5p-mediated P38 MAPK signaling	Tang et al.^342^
LINC01057	lncRNA	promotes mesenchymal differentiation	activating NF-κB	Tang et al.^343^
NEAT1	lncRNA	promotes malignant phenotypes and TMZ resistance in GBM stem cells	MAP3K1, as a direct target of let-7g-5p, is positively regulated by NEAT1	Bi et al.^230^
SNHG20	lncRNA	promotes tumorigenesis and cancer stemness	activating PI3K/Akt/mTOR signaling pathway	Gao et al.^344^
TP73-AS1	lncRNA	promotes TMZ resistance	regulation of the expression of metabolism-related genes and ALDH1A1	Mazor et al.^248^
PCAT1	lncRNA	PCAT1 knockdown restrained the sphere-formation ability, increased the apoptosis rate and DNA damage under radiation treatment	increase the expression of miR-129-5p and decrease the expression of HMGB1	Zhang et al.^276^
MALAT1	lncRNA	siRNA against MALAT1 sensitizes GBM to TMZ	–	Kim et al.^285^
SOX2OT	lncRNA	knockdown of SOX2OT inhibits the malignant biological behaviors	upregulating the expression of miR-194-5p and miR-122	Su et al.^294^
TALNEC2	miRNA	increased tumorigenic potential of GSCs and their resistance to radiation	downregulation of miR-21 and miR-191	Gao et al. and Brodie et al.^141^^,^^278^
miR-103a	miRNA	decreased the radioresistance capability	suppressing the FGF2-XRCC3 axis	Gu et al.^345^
miR-139	miRNA	inhibitory functions on GSC stemness and tumorigenesis	inhibiting Wnt/β-catenin signalling	Li et al.^202^
miR-27a-5p	miRNA	enhanced the sensitivity of glioma stem cells to radiotherapy	shFOSL1-inhibited miR-27a-5p expression	Li et al.^346^
miR-944	miRNA	reduces glioma growth and angiogenesis	inhibiting AKT/ERK signalling	Jiang et al.^216^
miR-128, miR-302a	miRNA	enhances senescence-associated cytotoxicity of axitinib to overcome drug resistance	–	Cardoso et al.^347^
miR-30b-3p	miRNA	confer TMZ resistance	directly targeting RHOB	Yin et al.^156^
miR-146b-5p	miRNA	suppresses the malignant phenotype	miR-146b-5p inhibited SMARCA5 expression and inactivated a TGF-β pathway	Wang et al.^127^
mir-370-3p	miRNA	inhibiting glioma cell growth, migration, and invasion	targeting the NEAT1, HMGA2, and HIF1A	Lulli et al.^140^
miR-603	miRNA	simultaneously promoted the CSC state and upregulated DNA repair to promote acquired resistance	targeting IGF1 and IGF1R	Ramakrishnan et al.^136^
miR-27a-3p, miR-22-3p, miR-221-3p	miRNA	exacerbated radiotherapy resistance	targeting CHD7	Zhang et al.^348^
miR-486-5p	miRNA	enhanced the self-renewal capacity	miR-486-5p as a Sox2-induced miRNA that targets the tumor-suppressor genes PTEN and FoxO1	Lopez-Bertoni et al.^134^
miR-30a	miRNA	suppresses self-renewal and tumorigenicity	blocking the NT5E-dependent Akt signaling pathway by targeting the NT5E	Peng et al.^124^
miR-124	miRNA	promotes a stem-like to neuronal transition, with reduced tumorigenicity and increased radiation sensitivity	targeting the SOX9 and inhibition of ERK1/2	Sabelström et al.^349^
miR-181d	miRNA	interferes in the GBM CSC response to treatment with TMZ and ionizing radiation	miR-181d associated with the methylation status of the MGMT	Lizarte Neto et al.^350^
miR-93	miRNA	enhanced the activity of IR and TMZ against GSCs	simultaneous inhibition of multiple autophagy regulators, including BECN1/Beclin 1, ATG5, ATG4B, and SQSTM1/p62	Huang et al.^121^
miR-7-5p	miRNA	suppresses stemness and enhances TMZ sensitivity of drug-resistant GBM	targeting Yin Yang 1 (YY1)	Jia et al.^142^
miR-186	miRNA	reverses cisplatin resistance and inhibits the formation of the GBM	degrading Yin Yang 1	Li et al.^351^
miR-29a	miRNA	improved sensitivity to cisplatin	–	Yang et al.^352^
miR-132	miRNA	induces TMZ resistance and promotes the formation of CSC phenotypes	targeting TUSC3	Cheng et al.^353^
miR-223	miRNA	increase the sensitivity of glioma to TMZ	regulating PI3K/Akt signaling pathway	Huang et al.^165^
let-7g-5p	miRNA	inhibits epithelial-mesenchymal transition consistent with reduction of glioma stem cell phenotypes	targeting VSIG4	Zhang et al.^225^
miR-146b-5p	miRNA	attenuates stemness and radioresistance	targeting HuR/lincRNA-p21/β-catenin pathway	Yang et al.^153^
miR-218-5p	miRNA	inhibits the stem cell properties and invasive ability	reduced stem cell marker (A2B5, nestin, PLAGL2, ALDH1 and Sox2) expression	Wu et al.^196^
miR-125b	miRNA	sensitize TMZ-induced anti-glioma stem cancer effects	inactivation of Wnt/β-catenin signaling pathway	Shi et al.^354^
miR-153	miRNA	decreased radioresistance and stemness	targeting Nrf-2/GPx1/ROS pathway	Yang et al.^166^
miR-30	miRNA	promotes glioma stem cells	decreased the expression of suppressor of cytokine signaling 3 (SOCS3) expression	Che et al.^148^
miR-210	miRNA	miR-210 knockdown decreases hypoxic glioma stem cells stemness and radioresistance	–	Yang et al.^355^
miR-455-3p	miRNA	TMZ resistance	–	Tezcan et al.^176^
miR-125b	miRNA	enhance the chemosensitivity of GBM stem cells to TMZ	targeting Bak1	Chen et al.^177^
miR-125b	miRNA	inhibition of miR-125b enhance sensitivity of GSCs to TMZ	targeting PIAS3	Shi et al.^209^
miR-17	miRNA	decreased cell proliferation and drug resistance	repress MDM2	Li and Yang^356^
miR-23b	miRNA	enhanced the sensitivity to TMZ	–	Geng et al.^172^
miR-145	miRNA	reduced chemoradioresistance	targeting Oct4 and Sox2	Yang et al.^357^
miR-125b-2	miRNA	resistance to TMZ	mitochondrial pathway of apoptosis	Chan et al.^158^
miR-9	miRNA	suppression of miR-9 confer stemness potential and chemoresistance	induces SOX2	Jeon et al.^358^
miR-328	miRNA	decrease the chemoresistance	targeting ABCG2	Li et al.^359^

## Availability of data and material

The primary data for this study are available from the authors on request.
